# Osseointegration of zirconia implants compared with titanium: an *in vivo *study

**DOI:** 10.1186/1746-160X-4-30

**Published:** 2008-12-11

**Authors:** Rita Depprich, Holger Zipprich, Michelle Ommerborn, Christian Naujoks, Hans-Peter Wiesmann, Sirichai Kiattavorncharoen, Hans-Christoph Lauer, Ulrich Meyer, Norbert R Kübler, Jörg Handschel

**Affiliations:** 1 Department of Cranio- and Maxillofacial Surgery, Heinrich-Heine-University, Düsseldorf, Germany; 2 Department of Prosthetic Dentistry, Section of Materials Sciences, Johann Wolfgang Goethe University, Frankfurt, Germany; 3 Department of Operative and Preventive Dentistry and Endodontics, Heinrich-Heine-University, Düsseldorf, Germany; 4 Department of Cranio- and Maxillofacial Surgery, Westfalian Wilhelms-University, Münster, Germany; 5 Department of Oral and Maxillo-Facial Surgery, Mahidol University, Bangkok, Thailand

## Abstract

**Background:**

Titanium and titanium alloys are widely used for fabrication of dental implants. Since the material composition and the surface topography of a biomaterial play a fundamental role in osseointegration, various chemical and physical surface modifications have been developed to improve osseous healing. Zirconia-based implants were introduced into dental implantology as an altenative to titanium implants. Zirconia seems to be a suitable implant material because of its tooth-like colour, its mechanical properties and its biocompatibility. As the osseointegration of zirconia implants has not been extensively investigated, the aim of this study was to compare the osseous healing of zirconia implants with titanium implants which have a roughened surface but otherwise similar implant geometries.

**Methods:**

Forty-eight zirconia and titanium implants were introduced into the tibia of 12 minipigs. After 1, 4 or 12 weeks, animals were sacrificed and specimens containing the implants were examined in terms of histological and ultrastructural techniques.

**Results:**

Histological results showed direct bone contact on the zirconia and titanium surfaces. Bone implant contact as measured by histomorphometry was slightly better on titanium than on zirconia surfaces. However, a statistically significant difference between the two groups was not observed.

**Conclusion:**

The results demonstrated that zirconia implants with modified surfaces result in an osseointegration which is comparable with that of titanium implants.

## Background

Since their introduction over 40 years ago, dental implants have become an established treatment modality that had revolutionized the concept of replacing missing teeth. The recent material of choice for manufacturing dental implants is commercially pure titanium, because of its excellent biocompatibilty and mechanical properties [1]. However, the gray colour of the titanium may be disadvantageous and give rise to esthetic problems, especially if the soft tissue situation is not optimal and the dark colour shines through the thin periimplant mucosa [2].

The success of endosseous implants is directly related to the principle of osseointegration, a process of implant-bone interaction that finally leads to bone-to-implant anchorage [3]. As the surface topography of a biomaterial has a major impact on osseointegration, various chemical and physical surface modifications have been developed to improve osseous healing of implants. Increased surface roughness of dental implants resulted in greater bone apposition [4] and reduced healing time [5].

Zirconia ceramics (yttrium-stabilized tetragonal poly-crystals) seem to be a suitable material for dental implants because of their tooth-like colour, their excellent mechanical properties and their good biocompatibility [6]. They have extensively been used as ball heads in total hip replacements with remarkable clinical outcomes [7]. Recent animal studies have also shown successful bone healing of dental zirconia implants under both unloaded and loaded conditions [2,8-10]. As the conventional fabrication of zirconia rods usually results in realtively smooth surfaces, only few studies have investigated rough surface modifications of zirconia implants. This is a critical aspect, since it has been already demonstrated that surface roughness and topography also influence osseointegration of zirconia implants [6,11-13].

In comparison with titanium implants, much less is known about the role played by surface modifications on the osseointegration of zirconia implants. Thus, the aim of the present study was to examine the osseous healing of zirconia implants with acid-etched surface structures in comparison with titanium implants.

## Methods

### Experimental animals

Twelve minipigs (> 5 years, average body weight 66.5 kg) were used in this study. The investigation was approved by the Animal Ethics Committee at the University of Düsseldorf, Germany. The animals were kept in small groups in purpose-designed sties and fed on a standard diet. Twelve hours before surgery animals were denied feed although water was accessible ad libitum.

### Implant system

Twenty-four screw-type zirconia implants (yttrium-stabilized tetragonal poly-crystals) with modified (acid-etched) surfaces (Ra = 0.598 μm, according to manufacturer) were used and compared to twenty-four implants made of commercially pure titanium with acid-etched surfaces (Ra = 1.77 μm, according to manufacturer). Implants were supplied by Konus Dental Implants (Bingen, Germany). All implants had the same macroscopic design with a standardized diameter of 3.5 mm and a length of 9 mm.

### Surgical procedure

All surgeries were performed under sterile conditions in a veterinary operating theatre. The animals were sedated by an intramuscular injection (10 mg/kg) of ketamine (Ketavet^®^, Pfizer, Karlsruhe, Germany), 1 ml atropine (Atropinsulfat Braun^®^, Braun, Melsungen, Germany) and 5 mg/kg azaperone (Stresnil^®^, Janssen-Cilag, Neuss, Germany).

Anesthesia was induced with an intravenous bolus of 3–5 ml thiopental (Thiopental inresa^®^, Inresa Arzneimittel, Freiburg, Germany) followed by intubation and maintenance of anaesthesia by inhalation of 1.5% isoflurane. For analgesia animals received 0.5 ml piritramide (Dipidolor^®^, Janssen-Cilag, Neuss, Germany). In the areas to be exposed to surgery, 5 ml of local anaesthesia [articain hydrochloride, (Ultracain^® ^DS, 1:200.000), Aventis, Frankfurt, Germany] was injected. The tibias were exposed by skin incisions and via fascial-periosteal flaps. Thereafter, four implants were placed in the tibia. The implant sites were sequentially enlarged with two drills according to the standard protocol of the manufacturer. Implants measuring 9 mm in length and 3.5 mm in diameter were inserted using continuous external sterile saline irrigation to minimize bone damage caused by overheating. At the surgical site, the skin and the fascia-periosteum were closed in separate layers with single resorbable sutures (Vicryl^®^2-0, Ethicon, Norderstedt, Germany). Perioperatively, the animals received amoxicillin (10 mg/kg KG) (Duphamox LA^®^, Fort Dodge, Würselen, Germany) as antibiotic and carprofen p.o. (4.4 mg/kg) (Rimadyl^®^, Pfizer, Karlsruhe, Germany) as antiphlogistic medication for three days. The animals were inspected after the first few postoperative days for signs of wound dehiscence or infection and, thereafter, weekly to assess general health. After 1, 4 or 12 weeks animals were sacrificed (4 minipigs each) with an overdose of pentobarbital (Eutha 77^®^, Essex Pharma, München, Germany) given intravenously. Following euthanasia, tibia block specimens containing the implants and surrounding tissues were dissected from the animals. The block samples were sectioned with a saw to remove unnecessary fragments of bone and soft tissue and were prepared for the subsequent investigations.

### Histological analyses

The implants were immediately fixed in 4% buffered formaldehyde for approximately one week. Then the specimens were dehydrated in a graded series of ethanol. Thereafter, samples were embedded in methyl metacrylate (Technovit^®^7200, Heraeus Kulzer, Dormagen, Germany). With the help of the cutting-grinding technique according to Donath, longitudinal sections were ground to about 20–40 μm for conventional microscopy (Exakt Apparatebau, Norderstedt, Germany). Two central histological sections of each implant were obtained and samples were stained with toluidine blue and Masson-Trichrome-Goldner. The slides were examined and photographed with a Leica DM 5000B (Leica Microsystems, Wetzlar, Germany) light microscope, equipped with a Leica DC 300F high resolution camera.

### Histomorphometry

Histomorphometric evaluation was performed after one central slice was chosen at 50-fold magnification using a digital camera. The software ImageJ 1.37v^® ^(open source: ) was used to measure the bone-to-implant contact (BIC) ratio, defined as the length of bone surface border in direct contact with the implant (× 100 (%)).

### Statistical analysis

All calculations were performed with the help of SPSS for Windows (SPSS Inc., Chicago, IL, USA). The results from the histomorphometric measurements were expressed as means ± standard deviations. The different treatment groups were compared using a Mann-Whitney *U *test. A p < 0.05 was set for significance.

## Results

**Figure 1:**
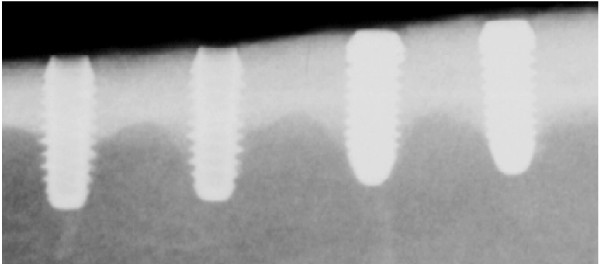
Radiograph showing titanium (left) and zirconia (right) implants inserted into the tibia of minipigs after 12 weeks of healing time.

**Figure 2:**
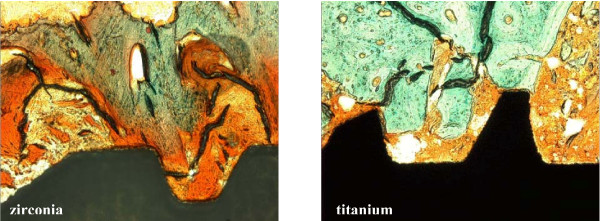
Micrograph showing matrix-rich regeneration tissue (orange) between the implant and bone (green). Zirconia implant (left), titanium implant (right) (Masson-Trichrome-Goldner, 100-fold).

**Figure 3:**
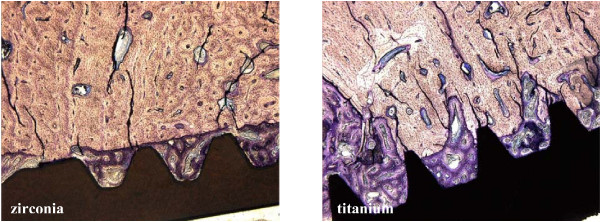
At 4 weeks after implantation, osteoid and woven bone were formed both on zirconia (left) and titanium implant surfaces (right) (toluidine blue, 50-fold).

**Figure 4:**
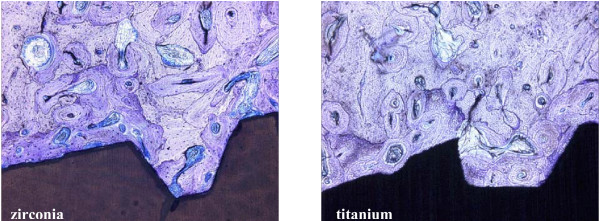
After 12 weeks of healing, mature lamellar bone is evident in intimate contact with the zirconia implant (left) and titanium implant (right) (toluidine blue, 100-fold).

The animals recovered well after surgery and no signs of infection were noted upon clinical examination at any time during the observation period (Figure 1). Light microscopical analysis demonstrated that matrix-rich regeneration tissue displaced the blood clot between the implant surface and the bone tissue in the first week after surgical procedure (Figure 2). After 4 weeks, mature regeneration tissue with formation of osteoid and woven bone was observed (Figure 3). Close contact of the bone to the implant was seen both on titanium and zirconia surfaces. Circumferential bone tissue formation was detectable on the zirconia implant surface. After 12 weeks of healing, hard tissue integration of the titanium as well as the zirconia implants was achieved. Mature lamellar bone in direct contact to the titanium and zirconia implants was found (Figure 4). No signs of inflammation were detected in any of the specimens. Histologically detectable minor differences between the zirconia and the titanium implants were no longer evident.

**Figure 5:**
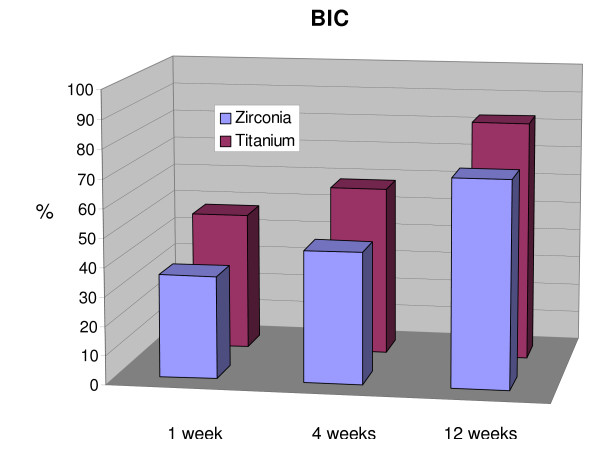
**Diagram depicting the increase in bone-to-implant contact (BIC) with time (1, 4, 12 weeks).** No statistical significance was detected between the two treatment groups (p < 0.05).

The bone-to-implant contact increased over the examination period for both zirconia and titanium implants (Figure 5). After 1 week of healing, the mean BIC was 35.3% ± 10.8 for the zirconia and 47.7% ± 9.1 for the titanium implants, respectively. After 4 weeks *in situ*, BIC of the zirconia implants averaged 45.3% ± 15.7 and 58.6% ± 9.5 for the titanium implants. After 12 weeks the BIC values were 71.4% ± 17.8 for the zirconia implants and 82.9% ± 10.7 for the titanium implants. There were no statistically significant differences observed betweeen the titanium and zirconia implants (p < 0.05) in regards to bone-to-implant contact after 1, 4 or 12 weeks.

## Discussion

Zirconia is a bioinert nonresorbable metal oxide that offers mechanical properties which are superior over other ceramic biomaterials, e.g. high fracture toughness and bending strength [14]. Because of its good chemical and material stability, high strength and resilience it seems to be a suitable material for dental application [7]. Its successful application in dentistry for fabricating endodontic posts and for crown and bridge restorations has been reported in several studies [15-17]. Especially because of its tooth-like colour, zirconia was suggested to be a desirable alternative material to titanium for the fabrication of dental implants. The results of the present study have shown that zirconia implants fabricated with a modified surface seem to be integrated into bone in a similar fashion as titanium.

After one week of healing, distinct gaps between the implant and the bone filled with matrix-rich regeneration tissue were observed in a few locations. After 4 weeks, woven bone, and after 12 weeks, lamellar bone, was visible in intimate contact with the implant surfaces. A loose connective tissue layer separating bone tissue and the zirconia surface as described by Sennerby et al. [6] previously, was not found in our samples.

Osseointegration of threaded zirconia implants has been recently investigated by Rothamel et al. [18]. They compared the osseous healing of zirconia implants with modified (machined and sand blasted) implant surfaces from polished commercially pure titanium. After 4 days of healing time, a distinct gap between bone tissue and the implant surface filled with remodelling blood clot was noticed. Two weeks after implantation, woven bone growing in the direction of the implant was observed, followed by the formation of lamellar bone after 28 days. When the healing period was nearly completed after 8 weeks, intimate contact of lamellar bone to the implant surface was evident. However, the barrier resulting from the original gap was still visible with many osteoblasts bridging the gap, which indicates a high biocompatibility of the used implant materials.

The results of the present study also showed an increasing BIC over the healing period. However, there were no statistically significant histomorphometrical differences observed between zirconia and titanium implants. This finding is in accordance with other animal studies which also failed to demonstrate differences between structured zirconia and titanium implant surfaces [2,6,8,12,19], likely secondary to the fact that zirconia is highly biocompatible. An average BIC > 60%, which was achieved after 4 weeks following implantation, had been reported by several authors [2,6,10,18]. The reported differences in BIC seem to be attributable to different animal models (dogs, monkeys, rabbits and minipigs) used for the experiments [2,9,20]. In order to establish standardized conditions for the histomorphometric analysis, implants were placed in the tibia since this bone has constant bone geometries over a longer distance. Therefore, the BIC only depends on the implant osseointegration and not on the bone features at the implantation site. In contrast to the results from a similar study [21], there were no detachment or separation of bone tissue and the zirconia surface with loose connective tissue detectable at any time.

The BIC measured in our study (45.3% after 4 weeks) showed similar results as demonstrated by Sennerby et al. [6]. The authors demonstrated a BIC of 36% for the non-modified zirconia implants and BICs of more than 45% for the zirconia implants with surface modification after 6 weeks of healing in the tibia of rabbits.

Scarano et al. [10] observed 68% BIC of the untreated zirconia implants after 4 weeks in the tibia of rabbits. After 6 months of unloaded healing in the mandibles of dogs, Dubruille et al. [9] measured a BIC of 65% for the zirconia implants compared with 68% of alumina implants and 54% of the titanium implants. The surface topography of the implants in these studies was not investigated. Kohal and coworkers [2] determined slightly higher BIC values after implant insertion into the maxillae of monkeys followed by 5 months of loaded healing (68% for sandblasted zirconia implants and 73% for sandblasted and acid-etched titanium implants). However, the surface topography was not measured or described. In the present study, a BIC of 71% for the acid-etched zirconia and 83% for acid-etched titanium implants were measured after 3 months of implant insertion.

It is well known that surface modifications can enhance bone integration of titanium implants in diverse animal models [22,23]. According to the results of several earlier experimental studies, surface roughness and topography influence osseointegration of zirconia implants to a greater extend [11-13]. Sennerby et al. [6] used a coating technique to receive porous surface modifications of the zirconia implants (nonmodified implants: Sa = 0.75 μm; modified implants: Sa = 0.93 μm, Sa = 1.24 μm, respectively). In spite of evident differences in surface roughness, there were no significant differences observed in the osseointegration (BIC or bone area filling in the threads) in the investigated implants. Only removal torque test values were significantly lower of the nonmodified zirconia implants compared with all other implant types. These results and the results of Scarano et al. [10], who used unmodified zirconia implants, indicate a considerable biocompatibility of zirconia implants, even without surface treatment.

In contrast to the study of Sennerby et al. [6], an acid-etching technique was used in this study to receive structured surface modification of zirconia implants. Surface modification by acid-etching is assumed to affect not only the microtopography, but also submicrometric and nanometric topography of implant materials. Sa or Ra values only refer to the average surface roughness. These values do not provide much information about the submicrometric and nanometric surface topography (Ra is the two-dimensional (2D) counterpart of the three-dimensional (3D) descriptor Sa. Both Ra and Sa reflect the arithmetic mean of the absolute values of the surface point departures from the mean plane within the sampling area [24]).

Submicrometric and nanometric topography determine cell reactions including cell orientation, changes in cell motility, cell adhesion and cell shape. Therefore these topographic features play an important role in the early state of osseointegration of dental implants [25]. In addition, differences in the physico-chemical properties of the material also affect cell responses [26].

The successful integration of zirconia implants into native bone tissue and comparable BIC was demonstrated in this study, however the used modified zirconia implants exhibited a considerable lower Ra value when compared to the titanium implants. Furthermore, the process of osseointegration of zirconia implants showed similarities to that known for titanium implants. This may be due to the fact that surface topography is not the only controlling factor when studying the biologic response to an implant material.

The results of earlier described studies implicate a good biocompatibilty even of unmodified zirconia implants. The submicrometric and nanometric topography of the zirconia surfaces produced by the acid-etched modification may have an additional synergistic effect on biocompatibilty and osseointegration of zirconia implants [27]. Further studies are needed to examine the influence of submicrometric and nanometric surface topography of zirconia implants to the osseointegration process.

## Conclusion

The results from our study suggest that zirconia implants with modified surfaces display features of osseointegration similar to those of titanium implants. These results are promising in using zirconia implants for dental application in the future.

## Competing interests

The authors declare that they have no competing interests.

## Authors' contributions

UM, CN, JH conceived the study design and performed surgery. HPW carried out the histological analysis and drafted the manuscript. RD participated in the design of the study, performed surgery and wrote the manuscript. HZ, MO, SK, HCL, NRK participated in the early preparation of the manuscript and contributed to write the revised version of the article. All authors read and approved the final manuscript.
